# Pathophysiology of Keratoconus: What Do We Know Today

**DOI:** 10.2174/1874364101711010252

**Published:** 2017-07-31

**Authors:** Uri Soiberman, James W. Foster, Albert S. Jun, Shukti Chakravarti

**Affiliations:** Cornea Division, Wilmer Eye Institute, Johns Hopkins University School of Medicine, Baltimore, USA

**Keywords:** Keratoconus, Pathogenesis, Morphology, Proteomics, Cytokine, Oxidative stress, TGF-β, Keratocytes, Corneal epithelium

## Abstract

Keratoconus is a common corneal ectasia that leads to progressive visual impairment. Numerous studies have shown abnormal protein expression patterns in keratoconic corneas. However, the specific mechanisms causing this disease remain ambiguous. This review aims to provide an update on morphological studies of the keratoconic cornea, relate these early studies with current findings from proteomic, biochemical and cell culture studies and to postulate possible pathogenic pathways.

## 1 INTRODUCTION

Keratoconus (KC) is a common ectasia of the cornea [1, 2]. Its reported incidence ranges between 50-230 per 100,000 and the estimated prevalence is 54.5:100,000. Clinically, the central or paracentral corneal stroma undergoes progressive thinning and loss of structural integrity that leads to bulging of the cornea, which gives the cornea its typical cone shape appearance in KC. The disease process in the cornea results in a myopic shift and irregular astigmatism that may cause significant impairment in visual function and may not be amenable to spectacle correction. Disease onset is usually at youth or adolescence with a gradual progression over the first few decades. KC may be an isolated finding or it may be associated with other systemic disorders or syndromes, such as: Down syndrome, Leber congenital amaurosis and connective tissue diseases (for instance: Ehlers Danlos, Marfan syndrome, *etc.*). Another association that has been made was atopy, and at least one study has shown elevated serum IgE levels in KC patients when compared to non KC patients [3]. The central corneal thickness (CCT), with respect to congenital and familial cases, may be a genetic trait as shown by a recent genome-wide association analyses that identified multiple loci which confer susceptibility to low CCT and keratoconus. [4]Specifically, two small nuclear polymorphisms were identified to confer high risk for developing KC: *FOXO1*and *FNDC3B*. *FOXO1* acts by regulating the TGF-β pathway, which by itself may be implicated in the pathogenesis of KC [5].

Additionally, evidence for the role of genetics in KC comes from prior evidence of familial aggregation of KC: unaffected relatives of KC patients may have abnormal videokeratography indices [6]. This finding is of consequence as these apparently unaffected relatives may have a similar shape to their corneas and thus may be undergoing a silent subclinical disease process. Additional evidence for genetics’ role in the pathogenesis of KC comes from population-based studies: it appears that the disease is more common in certain ethnicities than in others, for instance higher incidence is observed in Indians, Pakistanis and Saudis [7]. However, environmental factors should not be ignored and their improtance should be underscored as they may induce allergic diseases of the conjunctiva and subsequent eye rubbing, which has been associated with disease progression [8]. This review will focus on non-syndromic KC, and specifically on our current understanding of the complex pathogenesis of the disease.

## 2 CORNEA – STRUCTURE

The cornea is an avascular tissue that provides most of the refractive power of the eye [9]. It is composed of five layers: stratified epithelium and its basement membrane, Bowman’s layer, the stroma, Descemet’s membrane and the endothelium. For the purpose of this review, we will focus on the epithelium, the basement membrane, Bowman’s layer, and the stroma alone as evidence strongly suggests that these layers are implicated in the pathogenesis of KC.

The human corneal epithelium is approximately 50 microns thick and consists of 4-6 layers of non-keratinized stratified squamous epithelial cells. Its basement membrane is composed mainly of collagen type IV, laminin and entactin; and the major proteoglycan is perlecan [10].

Bowman’s layer is an ill-defined layer separating the epithelial basement membrane from the stroma. It is 8-12 microns thick and its collagen fibrils interweave into the stroma [9].

The stroma which comprises the bulk of the cornea is approximately 500 microns thick and is a highly ordered network of collagen fibrils and extracellular matrix. Collagen is the most common protein in the stroma, and type I collagen is the most common of these collagens. The major stromal proteoglycans are decorin, biglycan, lumican, keratocan and osteoglycin/mimecan [11].

## 3 MORPHOLOGY

All corneal layers may be affected in KC. Early histological studies and then confocal microscopy studies have shown consistent findings: epithelial changes were more apparent in severe KC, for instance: the cells were found to be elongated or spindle shaped in severe KC patients [12, 13]. The wing cell layer in severe KC had large, irregularly spaced nuclei and the mean diameter was significantly greater than in controls. Basal epithelial cells were shown to have variable changes in size compared to controls. Bowman’s layer was shown to be disrupted with an occasional protrusion of epithelial cells or keratocytes as was shown in prior histological studies. Stromal findings included degraded stroma, poorly differentiated keratocyte nuclei and stromal hyper-reflectivity. The former finding may indicate loss of the quiescent phenotype of the keratocytes whereas the latter finding is consistent with apical scarring observed clinically in severe KC eyes. In addition, the anterior keratocyte density was confirmed to be lower using confocal microscopy [14].

Evidences for structural abnormalities have also been demonstrated in electron microscopy studies: proteoglycans were shown to be more scant in some areas of KC corneas and the orientation of the collagen-proteoglycan network was also abnormal with the proteoglycans oriented parallel to the collagen fibrils instead of perpendicular, reflecting an alignment pattern more similarly associated with tendons rather than the cornea [15]. In an X-ray diffraction study, the mean collagen interfibrillar space in KC was shown to be similar to normal control corneas, so the hallmark finding of KC – apical stromal thinning – was not attributed to packing of the fibrils in the corneal apex but to fibril loss [16]. Another important finding from that study was that the fibril diameter was similar in both KC and controls, so changes in the size of the collagen fibril do not account for KC phenotype. An additional X-ray diffraction study demonstrated loss of normal orthogonal (peripheral) or isotropic (central) lamellar architecture in KC [17]. The abnormality in collagen organization was later shown to be present in both the anterior and posterior parts of the cornea, especially in the area of maximal steepening [18]. These changes may account for the biomechanical instability and weakening that are hallmarks of KC, especially because the anterior stroma confers the majority of the biomechanical strength to the cornea [19-23]. Interestingly, a mid-stromal transplantation of isolated Bowman layer was recently suggested as a method to flatten the anterior cornea in advanced KC [24, 25].

## 4 MOLECULAR CHANGES

### 4.1 Protein Changes in KC

Protein studies of keratoconus originate in the 1980s: these early studies that used SDS-PAGE electrophoresis showed that corneas inflicted with KC have a reduced amount of collagen [26]. A prior immunohistochemistry study had shown decreased staining of Collagen type XII [27]. However, these analytical methods were not perfectly capable to identify all types of collagen involved in the disease process. This problem was solved with contemporary proteomic studies that have shown specific decreases in collagens type XII, I and V. [28, 27]

Additional earlier evidence suggested that the culprit in KC is perhaps collagen degradation due to over-expression of gelatinases [29-32]. Other relatively recent studies also indicate elevated proteolytic activity in KC corneas. In one report, there was a 2.2 fold increase in mRNA of catalase, 1.8 fold increase in enzyme activity and a 1.8 fold decrease in tissue inhibitor of matrix metalloproteinase (TIMP)-1 [33]. Another recent report has shown increased tear expression of MMP-1,-3,-7, and -13 in KC compared to controls [34]. An interesting observation made by this study was that gelatinolytic and collagenolytic activity in KC patients was elevated compared to controls, but when the KC group was compared to KC patients who underwent corneal collagen cross-linking (CXL) – there was no difference. This suggests that cross-linking does not change the biology of the disease, but only induces tissue resistance to degradation, as expected. An additional study demonstrated that eye rubbing, which has been linked to disease progression in KC, can by itself cause an elevation in the tear concentrations of MMP-13 [35]. This could theoretically provide the explanation as to why stromal thinning progresses in KC patients who frequently rub their eyes. Despite this evidence for increased expression of proteolytic enzymes in KC, it is difficult to determine whether proteolytic activity is a primary defect in the disease or whether it reflects ongoing tissue repair and remodeling which is also associated with expression of proteolytic enzymes.

Other studies on molecular changes have suggested that inflammation may play a role in KC despite the fact that clinically, KC does not appear to be an inflammatory eye disease. In a pilot study that used ELISA techniques KC patients were shown to have elevated IL-6 and TNF-α compared to healthy controls [36]. Our group studied the tears of KC patients using a highly sensitive multiplex immune-bead assay [37]. This study demonstrated a significant reduction in the concentrations of IL-12, CCL5 and TNF-α in KC patients. Although not statistically significant, IL-6 levels were also elevated. Additionally, a trend was observed for elevation in IL-17. The development of highly sensitive mass spectrometry has made it possible to examine global differences in protein levels: mass spectrometry on proteins extracted from tear fluid indicated that KC patients have lower levels of zinc-α2-glycoprotein, immunoglobulin kappa chain and lactoferrin [38]. The latter is of particular interest because lactoferrin is thought to exert antimicrobial and anti-inflammatory effects, however lactoferrin levels have been shown to be reduced in numerous ocular surface diseases, such as: dry eye and contact lens induced papillary conjunctivitis [39]. Many KC patients are also contact lens users, and therefore the significance of the finding of reduced lactoferrin in KC remains unclear. An additional mass spectrometric proteomic study showed that MMP-1 was present only in KC subjects, contrary to healthy controls. [40, 37] A similar study from another group confirmed some of these findings: a recent cytokine microarray analysis study showed increased tear levels of IL-4, -5, -6, -8 and TNF-a in KC compared to controls [34]. IL-6 was the only cytokine significantly increased in KC when compared with the cross-linked (CXL) group. The CXL group had no change in proteases but had elevaed TNF-α compared to the control group.

The first proteomic study on the keratoconus cornea was performed on epithelial specimens alone [41]. The most dramatic changes were observed in the concentration of the epithelial differentiation marker cytokeratin 3 (*KRT3*) when compared to controls. Other proteins found in excess in KC were S100A4 and gelsolin; enolase 1 was down-regulated in KC. These are largely cytoskeleton-associated proteins. S100A4 is a calcium binding protein that can bind to multiple elements of the cytoskeleton. It stimulates cell migration and change in cell morphology. Gelsolin caps actin during its assembly. It is known to stimulate cell motility and has been linked to apoptosis. Enolase 1 that is mainly produced in the basal epithelial cells was reduced in KC, which suggests de-differentiation or a decreased proliferation of the basal cells. However, enolase 1 also has a role in glycolysis and in adhesion to the ECM [42, 43]. As a whole, these findings suggest that a cytoskeletal and/or cytoskeletal associated cell-to-cell or cell-to-ECM system may be faulty in KC corneal epithelium.

Additional evidence confirmed the findings of reduced enolase 1 production in the epithelium of KC cornea [44] The study also showed that the same was true for β-actin. Western blot analysis demonstrated broken fragments of these proteins that suggested protein degradation in KC, consistent with the above-mentioned findings of increased proteolysis in KC. As noted above, enolase 1 is a marker for epithelial basal cells and stem cells. The finding of enolase 1 downregulation in KC alludes to failure to repopulate the epithelial layer.

Another proteomic study of the corneal epithelium in KC demonstrated higher levels of lamin-A/C, keratin type I cytoskeletal 14 and 16, tubulin beta chain, heat shock protein cognate 71kDA protein, and S100-A4 [45]. Other findings were lower levels of transketolase, phosphoglycerate kinase 1, NADPH dehydrogenase 1, and 14-3-3 protein sigma. The latter is a protein involved in cellular signaling and interaction; cell cycle; cytoskeletal structure and intracellular trafficking [46]. In our iTRAQ mass spectrometric study of the cornea, the main epithelial proteins found to be increased were keratins (6A, 16), beta-hemoglobin, S100 calcium binding protein A8, calmodulin-like 3 protein, and others. [28] Vimentin, a marker of myofibroblastic transformation and epithelial to mesenchymal transformation during wound repair, was found to be increased. Significant decreases were observed in collagen type VI, a major structural component of microfibrils, lactotransferrin, cAMP-regulated phosphoprotein, collagen type VII alpha1 and collagen type XII that help to anchor epithelial cells to the underlying matrix, heparan sulfate proteoglycan 2 and others. Taken together, these observations underscore a structural cytoskeletal or a cell-basement membrane adhesion defect in the corneal epithelium in the disease process.

Given that the main clinically observable defect in KC is the thinning of the stroma, proteomic studies of KC stroma were warranted. In the same study described above, our group yielded the most comprehensive proteomic landscape of the keratoconic corneal stroma, identifying a total of 1,157 proteins. Decreases were detected in many structural extracellular matrix proteins of the corneal stroma such as, decorin, lumican and keratocan, collagen types I and V [28]. Decorin, lumican and keratocan are stromal proteoglycans that variably regulate collagen fibril structure, corneal transparency and thickness [47-49]. In addition, there were decreases in corneal crystallins that contribute to cellular transparency in the healthy cornea [50]. Elevated proteins in the stroma included adaptor-related protein complex 2 (AP2B1), immunoglobulin Lambda-like polypeptide 1(IGLL1), cell division cycle and apoptosis regulator 1 (CCAR1) and other regulators of cell cycle. These changes are consistent with altered cell proliferation and apoptosis-related programs. In addition, a large number of ribosomal proteins were elevated that suggest endoplasmic stress and perturbation of events during translation.

The cumulative proteomic findings suggest a generalized loss of the normal quiescent phenotype of the keratocytes and an increase in the abnormal protein expression, similarly to that associated with wound healing phenotype. The global decrease in different types of collagens is consistent with the clinical observation of debulked and thinned corneas in KC.

The aqueous humor provides nutrients to the posterior avascular cornea and has been recently studied by mass spectrometry [51]. Samples obtained from patients with keratoconus were also analyzed and revealed significant differences compared to controls [52]. Hemoglobin subunit beta, haptoglobin, plasma protease C1 inhibitor, basement membrane-specific heparin sulfate proteoglycan core protein, hemoglobin subunit delta, carbonic anhydrase 1 (and others) were all over-expressed in KC samples. Ceruloplasmin, apolipoprotein A2, actin cytoplasmic 2, latent transforming growth factor beta-binding protein 2 and Ig kappa chain V-I region EU as well as others were underexpressed. These findings underscore the complexity of KC and the diversity of proteomic findings in different studies.

## 5 PATHWAYS INVOLVED IN KC - HYPOTHESES

In view of the evidence obtained from proteomic studies, the involvement of a few potential pathways may be hypothesized:

### 5.1 Cytokine Dysregulation

Inflammatory cytokines have been shown to be over-expressed in KC. Our group has shown IL-17 over-expression in pooled samples from KC patients. Interestingly, the corneal epithelium is one of the targets of IL-17 and when human corneal epithelial cells lines are exposed to stress they can produce cytokines that promote Th-17 differentiation [53]. Despite this known association, it remains unclear whether it is pro-inflammatory cytokines that trigger the cascade that leads to the typical phenotype that we call KC or a dysregulated Th1, Th2 and Th17 response as suggested by our tear cytokine study [37].

### 5.2 Oxidative Stress

Oxidative stress has long been implicated in the pathogenesis of KC [54-57]. However, it was not until 2005 that elevated catalase RNA and activity were demonstrated in human KC corneas [33]. Catalase is the major pathway through which the cells dispose of excess hydrogen peroxide. Additional observations from that study included upregulations of numerous types of cathepsins, which by themselves can promote hydrogen peroxide formation, thereby feeding the oxidative cycle. This overall pro-oxidative environment was hypothesized to trigger the tissue destruction seen in KC. Additional evidence from recent studies provides more support of the hypothesis that oxidative stress may play a significant role in KC. A study of keratoconus corneas assessed their antioxidant capacity, nitrites, lipid peroxidation products and other oxidative stress markers [58]. Both KC and post-LASIK ectatic corneas had signs of elevated oxidative stress when compared to healthy controls, a finding consistent with prior studies in the field of KC. Another recent tear metabolite study has also shown that the ratio of lactate to pyruvate, a marker for oxidative stress, is elevated in KC [59]. Moreover, the ratio of reduced to oxidized glutathione was elevated in KC, which also supports the theory that oxidative stress plays a role in KC. Gene ontology analysis of that study suggested that oxidative stress may be the underlying mechanism that triggers apoptosis in KC. This may be a missing link between oxidative stress and keratocyte apoptosis that has been reported to occur in KC, particularly as the cornea is exposed to high levels of oxidative stress (atmospheric O_2_ levels and ambient ultraviolet irradiation).

### 5.3 Alterations in TGF-β and the Effect on The Extracellular Matrix (ECM)

The ECM is produced by keratocytes. This process is regulated, amongst others, by TGF-β signaling [60, 61] Not surprisingly, a known systemic disease, Marfan syndrome, which is associated with TGF-β pathway mutations may also manifest with KC [62] Other anterior segment pathologies besides KC have been described to occur more commonly in patients with Marfan syndrome, namely: megalocornea, corneal flattening or scleral thinning. The analogy of the aortic wall thinning and dilatation seen in Marfan syndrome and the corneal stromal thinning seen in KC is imperfect, but the association is enticing [63-67].

Two reports from our group have shown that the TGF-β axis may be dysfunctional in keratoconus. An immunohistochemical staining showed increased pSMAD 2 staining of the keratoconus corneal epithelium. In the second, cultured stromal cells from keratoconus corneas showed an abnormally increased phosphorylation of pSMAD1/5/8 – a signaling axis that is novel to the cornea [68, 69]. Another well recognized fact is that different isoforms of TGF-β have distinct effects on the extracellular matrix, but in one study an aberrant TGF-β1 response was noted in human keratoconus cell lines deployed in a 3D collagen gel [70]. In that study, increased ECM contraction and overexpression of collagen I and V were observed. An additional study demonstrated that some of the downstream messengers in the TGF-β axis may be abnormal in keratoconus: KC keratocyte cell lines had downregulated expression of SMAD6 and SMAD7 when compared to normal keratocyte cell lines [71]. Moreover, that study showed that the expression patterns of these secondary messengers were left unchanged despite stimulation with TGF-β isoforms, which in normal cells produced a more robust response. In a different study, human corneal stromal fibroblasts exposed to various isoforms of TGF-β demonstrated a differential fibrotic response, especially in cells exposed to TGF-β3, an isoform known for its anti-fibrosis properties [72]. If a similar response could be elicited in vivo, this may be a therapeutic target to prevent stromal scarring which is often observed in severe KC cases.

Altogether, these findings suggest that the TGF-β pathway may be an important effector route in KC and warrant further research.

## 6 CELLULAR RESPONSES IN KC

The hypothesis that the trigger for keratoconus lies in the epithelium was stipulated a few decades ago. The corneal epithelial basement membrane was shown to be irregular or absent in cases of severe KC using anti collagen type IV antibodies [73]. An abnormal architecture of the interface between the basal cell layer of the epithelium and the underlying stroma was demonstrated in KC corneas and specifically, the epithelium-stromal interface of the central cornea mirrored that of the normal limbal area [74]. Diminished collagen XII staining was demonstrated at the level of the epithelial basement membrane in KC corneas, a finding that was later confirmed in a proteomic study of KC [27, 28]. Collagen type XII is believed to anchor the epithelial layer to the underlying stroma. This evidence of epithelial-stromal interface abnormalities in KC does not by itself explain the classic corneal stromal thinning seen in KC, however, animal models of corneal epithelial injury demonstrated apoptosis of the underlying stromal keratocytes [75-77]. Keratocytes produce the ECM of the corneal stroma, but their phenotype is exquisitely dependent on their isolation from the environment, which may only be 60µm away. It is well understood that keratocytes are primed to apoptose upon insult to injury. This is hypothesized to be a mechanism to limit spread of viruses or other infections. If the area directly above the keratocytes is constantly depleted of barrier function due to lack of epithelial cells this may be sufficient to elucidate the apoptotic response of the keratocytes, contributing to loss of cell number. Subsequently an area persistently depleted of keratocytes may have collagen drop-out over time.

Keratocytes are specialized tissue fibroblasts that reside within the corneal stroma and produce most of the corneal stromal proteins [78-80]. In KC, keratocyte density is lower than in healthy controls. The role of keratocytes is to maintain the highly ordered extracellular matrix (ECM) of the corneal stroma, mainly: collagens I, III, V; the proteoglycans: keratocan, lumican and decorin as well as other proteins [11]. These ECM components, when properly arranged maintain a structurally stable cornea that is optically clear and possesses the desired refractive and biomechanical properties. For these reasons, keratocytes are likely to be implicated in KC, and their study in tissue cultures may be pivotal to the study of KC. However, when grown in culture keratocytes soon lose their corneal in situ morphology and transform into their fibroblastic phenotype unless grown in serum free media – then they remain dendritic and quiescent [11, 81].

Multiple in vivo confocal scanning microscopy studies have shown that keratocyte density in KC is lower than in normal subjects [14, 82, 83]. Keratocyte depletion in KC was reported to be the result of apoptosis, as shown by TUNEL immunohistochemistry staining of KC corneas [84]. This finding was later strengthened by a gene expression study that demonstrated differential expression of apoptosis genes in KC derived keratocytes, however the culture media in which the keratocytes were grown was rich in serum. Therefore, a potential bias is of concern, as the keratocytes may have assumed a fibroblastic phenotype typical for keratocytes grown in such conditions [85].

Our studies of keratocytes in culture also point to dysregulations of the TGFβ pathway in keratoconus. Additional studies are required to pinpoint alterations in specific intermediates in TGFβ signal propagations in KC keratocytes.

## DISCUSSION

Overall, it appears that KC may be caused by an aberrant tissue response to one or more unidentified stimuli that subsequently leads to keratocyte depletion or dysfunction, loss of collagen, and ultimately a biomechanically weak cornea with a degenerated ECM.

## CONCLUSION

Despite major leaps in methodology, the origin and pathogenesis of KC remain elusive. The findings of various studies are sometimes so dissimilar that their comparison and juxtaposition become quite challenging. The fact that KC is a spectrum of phenotypes ranging from mild to severe vision disabling disease does not simplify the researcher’s task either. Naturally, studies performed on end-stage cases are less likely to demonstrate similar findings as in early and active disease – which may explain some of the heterogeneity in the findings of different studies. Other factors that need to be considered are that the disease itself may be multifactorial – caused by multiple genetic mutations; it may be affected by environmental factors such as climate and geography; and it may also be the final phenotype or pathway of multiple processes occurring simultaneously at the level of the epithelium and the stroma. Ultimately research on keratoconus is striving to identify these multiple-gene environment interactions to better understand disease processes in individual patients and tailored care.
